# Want to Grow? Just Say Yes…Mostly

**DOI:** 10.1111/acem.70129

**Published:** 2025-08-18

**Authors:** Judd E. Hollander

**Affiliations:** ^1^ Senior Vice President of Healthcare Delivery Innovation, Jefferson Health, Department of Emergency Medicine Sidney Kimmel Medical College of Thomas Jefferson University Philadelphia Pennsylvania USA

In 2017, when I was honored to give the keynote address at the Society for Academic Emergency Medicine Annual Meeting, I focused on the future of emergency medicine; however, when speaking with 3000 mostly younger colleagues I felt like I should impart some pearls of wisdom I learned over my career (Figure 1).

**FIGURE 1:**
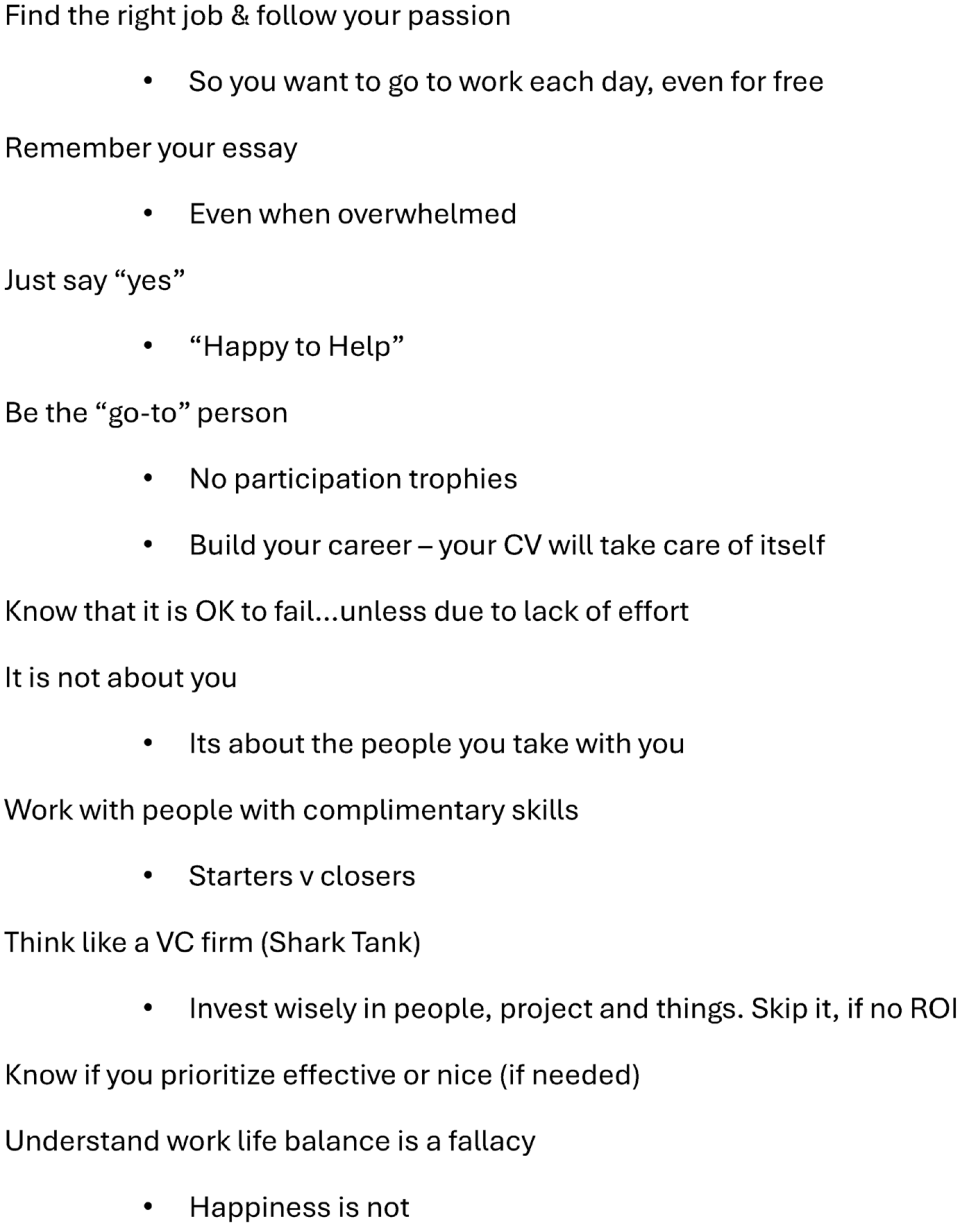
Recommendations from 2017 keynote at Society for Academic Emergency Medicine Annual Meeting.

These tips are rooted in two of my fundamental beliefs. (1) Your career will evolve. Data on medical school matriculants shows that the predicted specialty at the time of entrance is often quite different than that on match day. Similarly, your career goals are likely to change based upon opportunities that come your way, and (2) work‐life balance does not really exist. Happiness does. Some people find more happiness at work and others find it at home. You cannot be 100% at work and 100% at home. You need to decide on your own personal priorities, recognizing it may change over time.

It is helpful to have a “true north” or your own set of guiding principles. Ideally, the job you selected will enable you to wake up each morning ready to follow your passion. Think about the essay you wrote for medical school (or residency), where you were really clear about your goals. Try to continue to be that person. It can restore your sense of purpose. Although I don't know most of you reading this, I am betting that essay was about helping others. Retaining that focus and passion will help prevent burn‐out, allowing you to “just say yes.”

Perhaps one of the most important questions you need to contemplate is how you can best position yourself to evolve, grow and re‐create yourself for the next 40 years. I would put forth that the answer is to “just say yes.” I never thought I would have a research career. I never thought I would end up in health system leadership designing, implementing and running an enterprise virtual care program. I just simply said yes to trying new things, did what I was asked and was willing to fail but always learned enough to make it worthwhile. I was “happy to help.”

Years ago, one of my colleagues, a renowned researcher, moved into a role with the federal government. I did not understand how he made the change. In a conversation in the halls of the SAEM annual meeting, he told me that he could have a larger influence on injury prevention by helping to create regulations to keep millions of people safe than by treating one patient at a time in the emergency department.

He kept his focus on helping others, while recognizing that not all of the “others” are patients. For me, job satisfaction is heavily tied to people I can help be successful. It is as much about colleagues and mentees as patients. Someday I will not be here, but I can help mentor and advance others who will be here after I retire.

Please lose the focus on building the curriculum vitae. Do not volunteer for things solely for that reason. Lines on the curriculum vitae do not matter. There are no participation trophies.

Get skills rather than letters. Extra degrees do not matter nearly as much as extra skills. I never got an MBA, but I studied finance and management. Build your skill set. Your career will take care of itself.

Too often people give advice to mentees about how they should protect themselves and “just say no.” Say “no” one too many times and no one is coming back with new offers that you might have wished you received. That time might come to say “no”, but it isn't until you have already become a reliable “go‐to” person.

If you are not sure how to select the right projects, I recommend you watch Shark Tank. Investing time and money wisely, whether it be in a business, a research study, or a career requires the same considerations—does the project solve an unmet need? Does the team have the right skill set to accomplish it? What is the likelihood of success? What is the return on investment for the work? Evaluate these decision points when assessing offers. Invest wisely in people, projects, and things.

Saying yes does not mean you need to do all the work on your own. It is critical that you work with the right partners on the right projects. Personally, I look for two types of people—those willing to push back against me and those with complimentary skills sets. If you are an “in‐the‐weeds” detail‐oriented person, you might like to partner with a big picture strategic thinker. If you are great at starting projects but suffer from imperfect follow through, partner with a “closer”, someone who can pull together and complete the work you started. The short stop does not play first base or vice versa. Maximizing the skill set of your team with the right teammates allows you to “just say yes.”

The beautiful thing about being the person who says yes is that people will come back to you over and over again. Eventually, you can begin to pick and choose when to say yes. When I began my work with SAEM, I volunteered to be on the program committee. I volunteered to take on sub‐committee work. Within a short time frame, I was co‐chairing the scientific subcommittee and shortly thereafter became Program Committee Chair and then a member of the Board of Directors and ultimately the SAEM President, all within 10 years. I accepted the offer to review articles for several journals and became Associate Editor of Academic Emergency Medicine and later Deputy Editor for Annals of Emergency Medicine. Not coincidentally, I also became a better writer and researcher.

I do tell my mentees to “just say yes” but you should not say yes to absolutely everything. You should be careful not to say yes and not perform the work. After years of saying yes, you can pick and choose when to say no. Some things are total drains on your time. It is my personal belief that writing promotion letters fits this category. They are sent to you by someone someplace where you are not employed, the candidate has already met all internal criteria, and they are overwhelmingly likely to get promoted. The expectation is you will summarize their CV and respond to a few specific questions. Why don't they only send requests for people who do *not* clearly meet criteria? Why don't they send you a single page that says “meets criteria” or “does not meet criteria”? I now have a template that basically says I have reviewed the file and recommend them for this promotion. This is one way to save time.

Some committee work is the same. You do have an obligation to be a good “citizen” at your own institution, and in some cases, you might bring value to the committee. For others, having 15–20 people on a committee seems more intended to build a curriculum vitae for promotion than to get any real wisdom out of the members. Participate actively in the groups where you can add wisdom, and politely move away from those where you make no real meaningful contributions. You can suggest your membership on an alternate committee, which still makes you a team player. You get to say “yes” (sort of) while better aligning your interest and skills within your own institution.

My final piece of advice is the most difficult to apply. Know if you prioritize being effective or nice when you cannot be both. I am not sure I have mastered this one, so I will not tell you how to achieve this, but it probably helps to think about it in advance. If you are an emergency physician, this is not terribly different than being a patient advocate for that “soft admission” that the inpatient team is trying to resist. You need to know which muscles to exercise when.

In conclusion, please think about saying yes more often. It opens doors and opportunities that lead to a very fulfilling life. You can say no, but it will not be long until people stop giving you opportunities. You will only have yourself to ask why.

## Data Availability

The author has nothing to report.

